# Disease-related protein co-expression networks are associated with the prognosis of resectable node-positive pancreatic ductal adenocarcinoma

**DOI:** 10.1038/s41598-022-19182-9

**Published:** 2022-08-29

**Authors:** Toshihide Nishimura, Tatsuyuki Takadate, Shimpei Maeda, Takashi Suzuki, Takashi Minowa, Tetsuya Fukuda, Yasuhiko Bando, Michiaki Unno

**Affiliations:** 1 Department of Translational Medicine Informatics, St. Marianna University School of Medicine, Kawasaki, Kanagawa 216-8511 Japan; 2 Department of Surgery, National Hospital Organization Sendai Medical Center, Sendai, Miyagi 983-8520 Japan; 3 Department of Surgery, Saitama Medical Center, Jichi Medical University, Shimotsuke, Tochigi 329-0498 Japan; 4 Department of Pathology and Histotechnology, Tohoku University Graduate School of Medicine, Sendai, Miyagi 980-8574 Japan; 5 Nanotechnology Innovation Station, National Institute for Materials Science, Tsukuba, Ibaraki Japan; 6 Biosys Technologies, Inc., Tokyo, Tokyo, 153-8904 Japan; 7 Department of Surgery, Tohoku University Graduate School of Medicine, Sendai, Miyagi 980-8574 Japan

**Keywords:** Biological techniques, Cancer, Cell biology, Computational biology and bioinformatics, Molecular biology, Stem cells, Systems biology, Biomarkers, Diseases, Gastroenterology, Medical research, Molecular medicine, Oncology, Risk factors

## Abstract

Pancreatic ductal adenocarcinoma (PDAC) is a multifactorial disease, the molecular profile of which remains unclear. This study aimed at unveiling the disease-related protein networks associated with different outcomes of resectable, node-positive PDAC cases. We assessed laser-microdissected cancerous cells from PDAC tissues of a poor outcome group (POG; *n* = 4) and a better outcome group (BOG; *n* = 4). Noncancerous pancreatic duct tissues (*n* = 5) were used as the reference. We identified four representative network modules by applying a weighted network correlation analysis to the obtained quantitative PDAC proteome datasets. Two network modules that were significant for POG were associated with the heat shock response to hypoxia-related stress; in the latter, a large involvement of the non-canonical Hedgehog pathway (regulated by GLI1), the internal ribosome entry site-mediated cap-independent translation, the inositol requiring enzyme 1-alpha (IRE1α)/X-box binding protein 1 pathway of the unfolding protein response (UPR), and the aerobic glycolysis was observed. By contrast, the BOG characteristic module was involved in the inactivation of the UPR pathway via the synoviolin 1-dependent proteasomal degradation of IRE1α, the activation of SOX2, and the loss of PALB2 (partner and localizer of BRCA2) function, all potentially suppressing malignant tumor development. Our findings might facilitate future therapeutic strategies for PDAC.

## Introduction

Pancreatic cancer is the seventh leading cause of cancer-related deaths worldwide and the most lethal of the malignant ones, with a 5-year survival rate of 9%^1,2^. Pancreatic adenocarcinoma is the most common type of pancreatic cancer (85% of the cases) arising from the exocrine glands of the pancreas, with pancreatic ductal adenocarcinoma (PDAC) being its most common form^3^. Very few PDAC patients (less than 20%) receive curative radical surgical resection, and even in these patients, the recurrence rate is as high as 85%^4^. Patients with the Union for International Cancer Control (UICC) stage IIA (extends to the surrounding organs; node-negative) and IIB (extends to the surrounding organs; node-positive) PDAC are most frequently regarded as candidates for the undertaking of a curative resection^5–7^. UICC stage IIB PDAC still contains wide heterogeneity. Since stage IIB pancreatic cancer is the group with the worst treatment outcome (lymph node metastasis positive) among resectable pancreatic cancers, it is pivotal to unveil the molecular profiles between two PDAC groups with the same histological IIB grade and with the same clinical stage that is treated similarly but resulted differently in better or poor outcomes.

**Figure 1:**
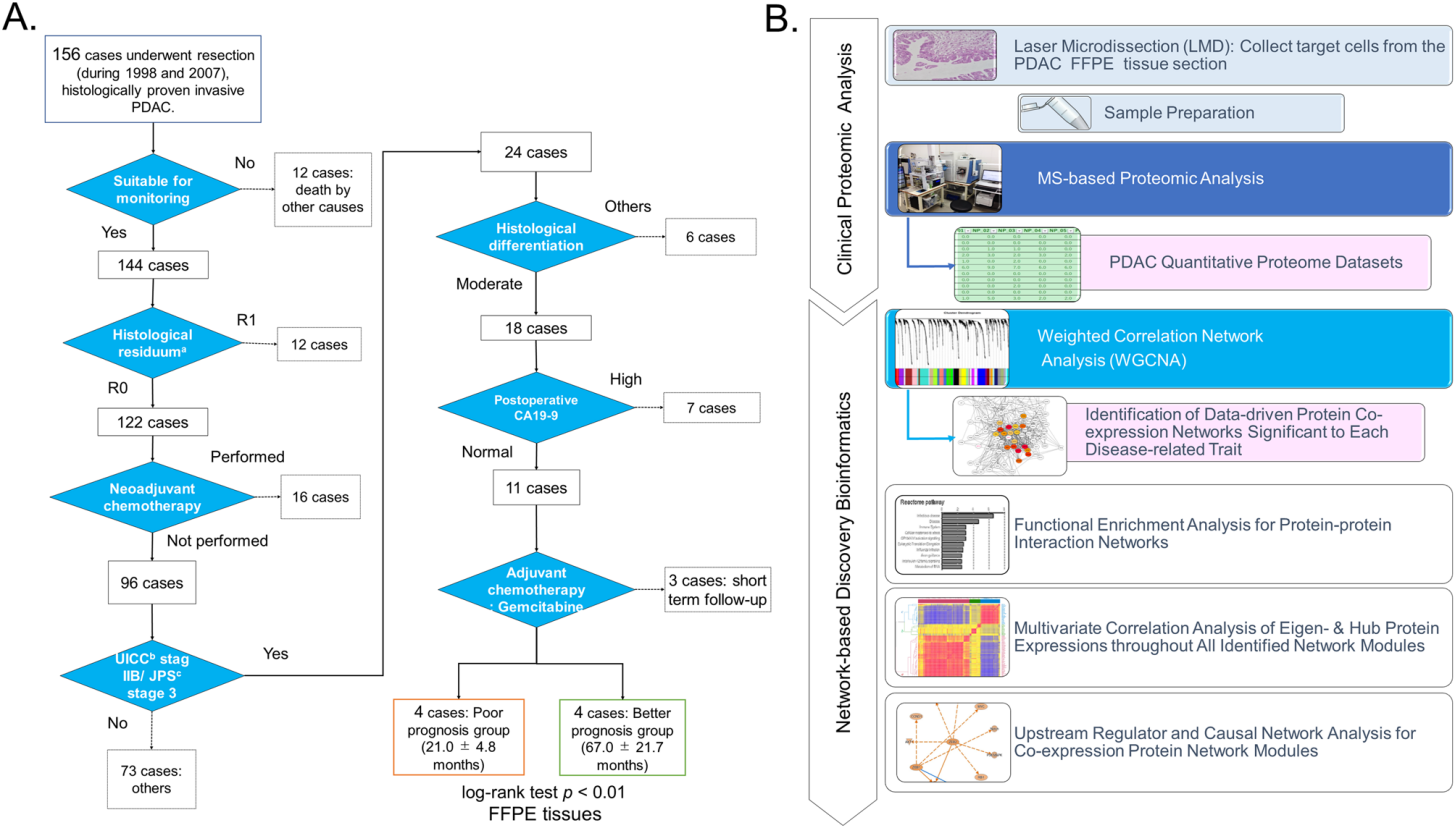
(**A**) Strategy followed for the selection of two PDAC groups that have the same histological grade within a clinical-stage and are treated similarly but result in different outcomes. (**B**) The workflow of the network-based discovery bioinformatic analysis following the clinical proteomic analysis. Notes: ^a^histological residuum R0 shows microscopically negative margins; ^b^Unio Internationalis Contra Cancrum/Union for International Cancer Control (UICC) stage IIB: cancer has spread to nearby lymph nodes and may also have spread to adjacent tissues and organs; ^c^Japan Pancreas Society (JPS) stage 3: cancer has not spread into the portal vein, the extra-pancreatic nerve plexus or other organs in UICC stage IIB.

High-accuracy mass spectrometry (MS)-based proteomics has advanced shotgun sequencing and the quantitative analysis of proteins expressed in clinical specimens. The obtained quantitative proteome data can be used to identify key disease-related proteins and therapeutic targets^8^. We have, herein, adopted label-free spectral counting-based semiquantitative MS-based proteomics that was applied on target cells obtained from formalin-fixed paraffin-embedded (FFPE) PDAC tumor specimens by using laser microdissection. The present study aimed at identifying the protein co-expression networks that are significantly associated with a poor and better clinical outcome in patients with resectable, node-positive (UICC stage IIB/JPS stage III) PDAC. Weighted gene co-expression network analysis (WGCNA)^9,10^ was applied to quantitative proteome datasets of PDAC. Our case selection strategy and the workflow of the employed network-based discovery bioinformatics analysis (taking place after the MS-based proteomic analysis) are presented in Fig. 1.

## Results

### Proteome datasets of node-positive PDAC

MS-based proteomic analysis was conducted on FFPE tissue specimens of resectable, node-positive PDAC patients with poor (*n* = 4) and better (*n* = 4) outcomes, based on the patients’ survival duration, while, essentially, their clinicopathological backgrounds were otherwise the same. Noncancerous pancreatic duct (NPD) tissue specimens (*n* = 5) were also analyzed as a reference and were obtained from the bile duct and ampulla of Vater carcinoma patients who underwent pancreaticoduodenectomy and whose cancer cells did not extend into the pancreas.

A total of 156 histologically diagnosed PDAC cases underwent pancreatectomy at the Tohoku University Hospital between January 1998 and December 2007. PDAC and TNM were classified according to the JPS (6th edition) and UICC (7th edition) at the time when PDAC cases were selected^11^. Our case selection strategy (Fig. 1A) was the following: (i) we selected 144 cases (by excluding those with perioperative mortality) suitable for monitoring; (ii) we chose 103 cases with microscopically complete resection (R0) and no evidence of para-aortic lymph node metastasis; (iii) among the 87 identified cases that received no neoadjuvant chemotherapy, we focused on those with standardized known prognostic factors, such as pathological stage, histological differentiation, postoperative carbohydrate antigen 19-9 levels, and adjuvant chemotherapy; and (iv) we finally chose eight cases that were subsequently divided into two groups based on the significant difference in their postoperative average survival time as calculated using the Kaplan–Meier method (the log-rank test; *p* = 0.0067): the poor outcome group (POG; *n* = 4; 21.0 ± 4.8 months) and the better outcome group (BOG; *n* = 4; 67.0 ± 21.7 months) (Table 1).

**Table 1:** Clinicopathological information regarding the recruited patients.

Patient group	Sample ID	Age	Gender	Tumor location	Clinical TNM classification*	UICC stage*	JPS stage*	Differentiation	Residuum	Postoperative CA19-9	Adjuvant chemotherapy	Postoperative survival months
Noncancerous pancreatic duct (NPD) (n = 5)	NPD-01	70	M	Bile duct								
	NPD-02	73	F	Bile duct								
	NPD-03	67	M	Bile duct								
	NPD-04	69	M	Vater								
	NPD-05	75	F	Vater								
			M(60%) / F (40%)									
	Average ± SD	70.8 ± 3.2										
Poor outcome group (POG) (n = 4)	POG-01	53	M	Head	T3N1M0	IIB	3	mod	R0	< 37 U/ml	Gemcitabine	15.9
	POG-03	67	M	Head	T3N1M0	IIB	3	mod	R0	< 37 U/ml	Gemcitabine	17.9
	POG-04	68	M	Head	T3N1M0	IIB	3	mod	R0	< 37 U/ml	Gemcitabine	24.5
	POG-06	79	F	Head	T3N1M0	IIB	3	mod	R0	< 37 U/ml	Gemcitabine	25.6
			M(75%) / F (25%)									
	Average ± SD	64.0 ± 10.7										21.0 ± 4.8
Better outcome group (BOG) (n = 4)	BOG-02	74	M	Head	T3N1M0	IIB	3	mod	R0	< 37 U/ml	Gemcitabine	42.8
	BOG-03	57	M	Head	T3N1M0	IIB	3	mod	R0	< 37 U/ml	Gemcitabine	56.6
	BOG-07	48	F	Head	T3N1M0	IIB	3	mod	R0	< 37 U/ml	Gemcitabine	76.2
	BOG-08	74	M	Head	T3N1M0	IIB	3	mod	R0	< 37 U/ml	Gemcitabine	92.3
			M(75%) / F (25%)									
	Average ± SD	65.4 ± 12.9										67.0 ± 21.7
Group comparison	t-test *p* value =	0.691									Log-rank test *p* value =	0.0067

*The JPS (6th edition) and UICC (7th edition) were used at the time when PDAC cases were selected^11^.

**Figure 2:**
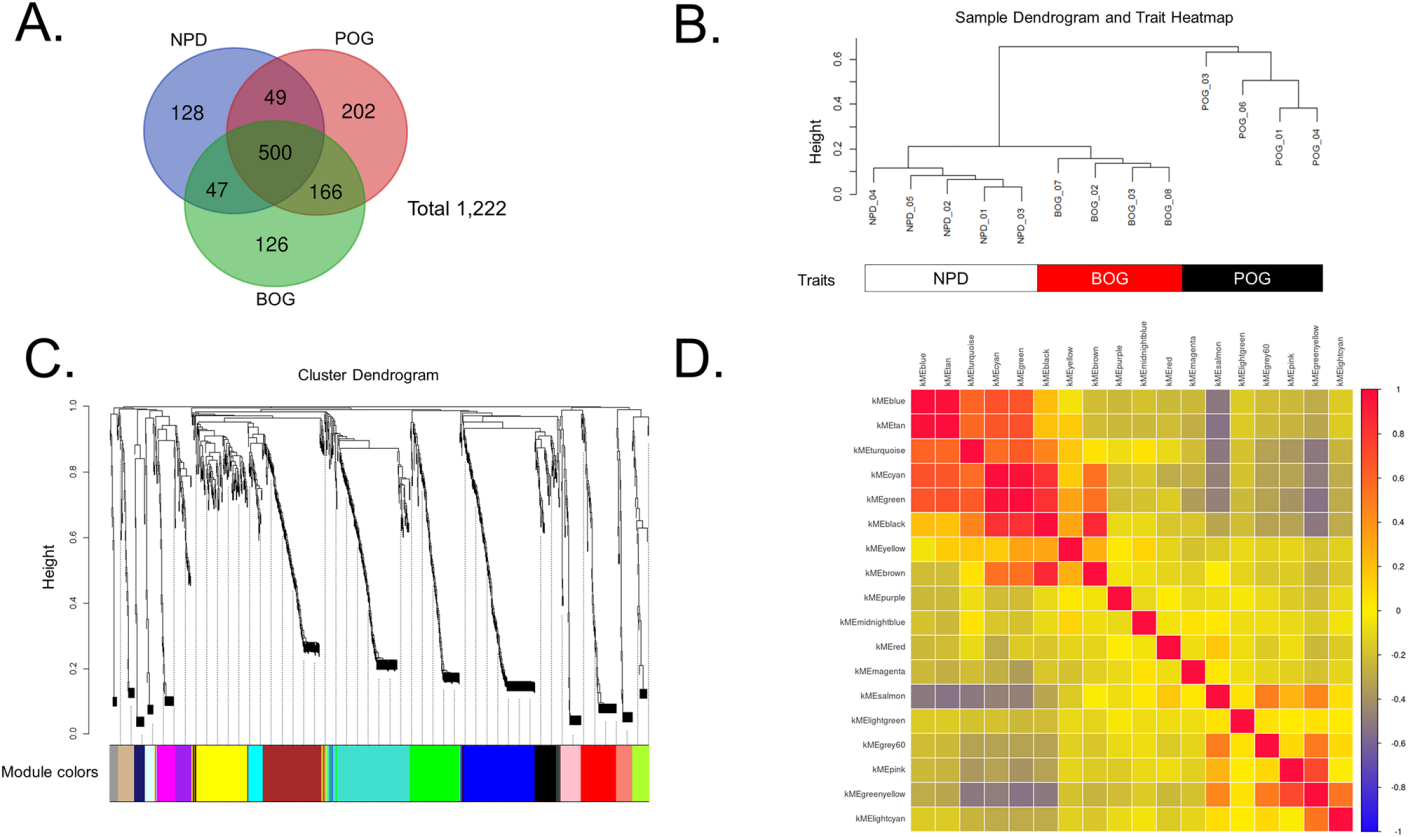
Gene modules identified by weighted gene co-expression network analysis (WGCNA). (**A**) Venn map of the identified proteins. NPD, the noncancerous pancreatic duct; POG, the poor outcome group; BOG, the better outcome group. (**B**) Sample dendrogram and trait heatmap. (**C**) Protein dendrogram obtained by clustering dissimilarity according to the topological overlap with the corresponding module; the colored rows correspond with the 18 modules identified by dissimilarity according to the topological overlap. (**D**) Pairwise correlations between the modules in the connectivity measure (K_ME_) of the module eigen-protein (correlation coefficient: Pearson; heatmap order: eigenvectors; agglomeration method: complete; the number of clusters: 3).

A total of 1,222 proteins were identified, of which 500 (40.9%) were commonly expressed in the cancerous cells of the POG, the BOG, and the NPD Group. Moreover, 202 (16.5%), 126 (10.3%), and 128 (10.5%) proteins were unique to the POG, the BOG, and the NPD Group, respectively (Fig. 2A).

### Data-driven co-expression protein network by WGCNA

The hierarchical clustering of the samples according to protein abundance exhibited a clear correlation with the three traits of the POG, the BOG, and the NPD Group (Fig. 2B), in which both the NPD group and the BOG were found to be closely clustered. In particular, 18 protein modules were identified by clustering all identified proteins and constructing weighted protein co-expression networks (Fig. 2C). The WGCNA analysis was performed with a soft threshold power of “8” that was selected to approximate a scale-free topology, a minimum module size of “10,” and a module detection sensitivity (*deepSplit*) of “4.” Correlations between the resultant modules and the traits were obtained to identify the protein modules that were significant to the respective traits. Pairwise correlations between the modules in the connectivity measure (KME) of the module eigen-protein are presented in Fig. 2D.

We identified three significant modules with high and/or moderate correlations (*r* > 0.5) and statistical significances (multiple testing correction using the Benjamini–Hochberg method: *q* < 0.05) with clinical traits (Fig. S1). The WM5 module (green-yellow; *r* = 0.88, *q* = 0.001) was most significantly correlated with the NPD Group. Both the WM7 (black; *r* = 0.76, *q* = 0.025) and WM11 (green; *r* = 0.94, *q* = 2.56 × 10^−5^) modules were significantly correlated with POG. Trait correlation analysis often tends to overlook important modules. The four identified WGCNA modules − WM15 (purple), WM16 (red), WM17 (magenta), and WM18 (midnight-blue)—correlated moderately (0.4 < *r* ≤ 0.5) with the BOG, but none of these correlations were found to be significant (*q* > 0.05). The statistical over-representative analysis could help in evaluating potential key WGCNA modules with identified proteins uniquely expressed for each trait. The overlaps of the WGCNA-derived modules with 202, 126, and 128 proteins that were found to be uniquely expressed in the POG, the BOG, and the NPD Group, respectively (Fig. 2A), were subsequently assessed by using the over-representation test. Among the four BOG characteristic modules (WM15, WM16, WM17, and WM18), WM16 (red) was the most significant (*r* = 0.49; overlapping *q* = 3.49 × 10^−39^) (Fig. S1).

### Functional enrichment analysis of the protein–protein interaction (PPI) networks

**Figure 3:**
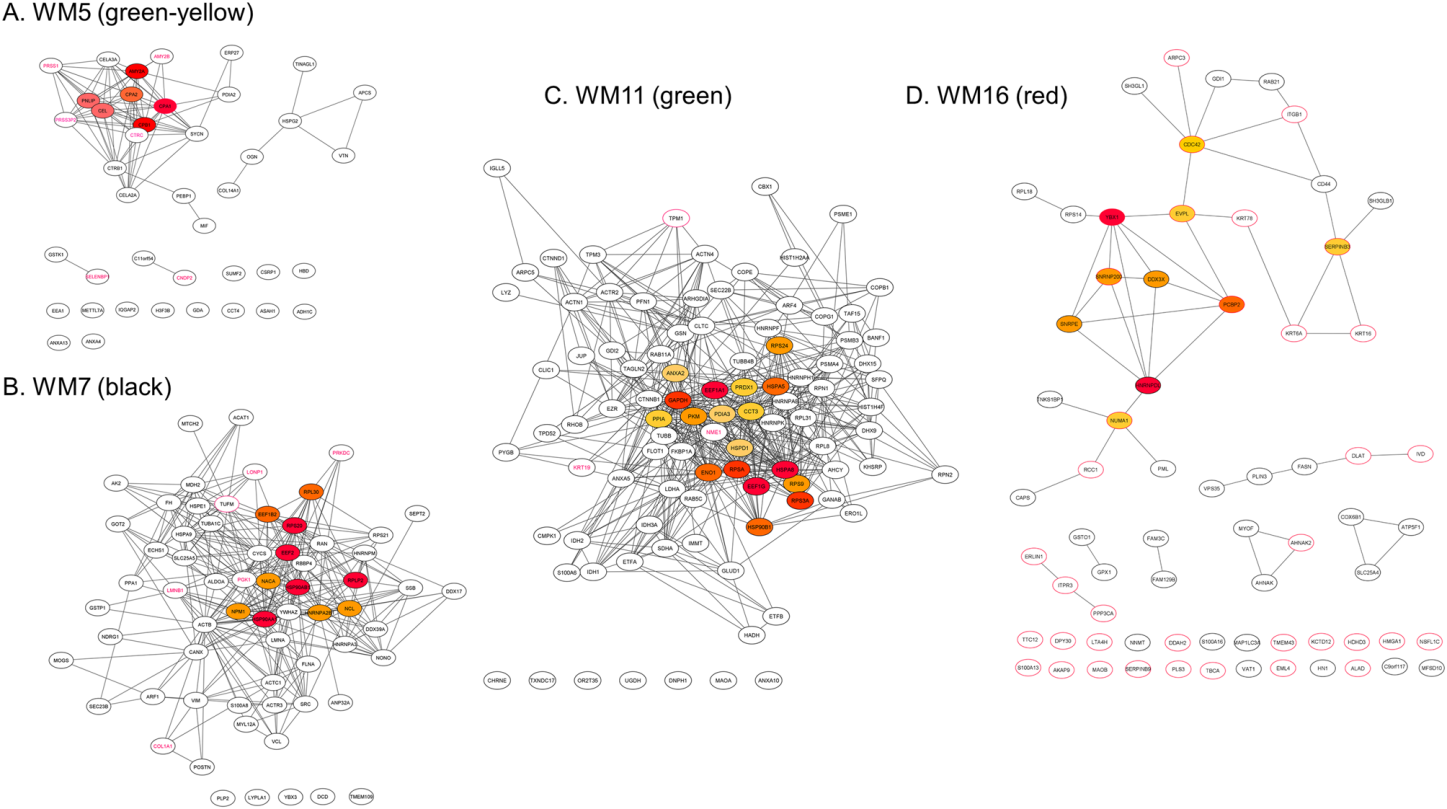
Data-driven protein co-expression networks: WM5 (green-yellow) (**A**), WM7 (black) (**B**), WM11 (green) (**C**), and WM16 (red) (**D**) modules. Circle nodes with a red border and with a fill color ranging from red to orange represent the eigen proteins and/or hub proteins, respectively, for each module. Circle nodes with a red letter also indicate key proteins in the network modules. The top 10 pathways enriched for the protein core networks obtained for biological process (GO), KEGG pathways, and Reactome pathways are presented in Fig. S2 in an order of significance defined by the *q*-value.

The Search Tool for the Retrieval of Interacting Genes/Proteins (STRING) database^12^ was used to generate the human PPI networks for the four WGCNA network modules: WM5 was significant to the NPD Group trait, WM7 and WM11 were significant to the POG trait, and WM16 was significant to the BOG trait. Those PPI networks were reconstructed by using the Cytoscape (version 3.8.2) software (Institute for Systems Biology, Seattle, WA, USA; https://cytoscape.org/)^13^ (Fig. 3). Top hub proteins were determined by using the *cytoHubba* plugin with maximal clique centrality (MCC)^14^. In data-driven protein co-expression networks, eigen-proteins are indicated in red borders, hub proteins in red to orange fill colors, and some key proteins are denoted in red letters. Top pathway enrichment results for the WGCNA modules are presented in Fig. S2.

The pathways enriched for the WGCNA module that was significant to the NPD Group (Fig. 3A), included the following: (i) digestion as a biological process (GO), (ii) pancreatic secretion as well as protein digestion and absorption as KEGG pathways, and (iii) digestion as a Reactome pathway (Fig. S2). The eigen-protein carboxypeptidase A1 (CPA1) is a member of the carboxypeptidase A family of zinc metalloproteases, the mutations of which have been linked to chronic and hereditary pancreatitis^15^. The hub proteins were CPA1, carboxypeptidase B (CPB1), carboxypeptidase A2 (CPA2), pancreatic amylase alpha 2A (AMY2A), carboxyl ester lipase, and pancreatic lipase (PNLIP), while other key proteins included cationic trypsinogen (PRSS1; trypsin 1), chymotrypsin-C (CTRC), amylase alpha 2B (AMY2B), PRSS3 pseudogene 2 (PRSS3P2; trypsin-2), selenium binding protein 1 (SELENBP1), and carnosine dipeptidase 2 (CNDP2). A set of pancreatic tissue-specific proteins (CPA1, CPA2, CPB1, and CTRC) are carboxypeptidase family members secreted by the pancreas and are downregulated in pancreatic cancer^16^. In Japan, the cumulative incidence of pancreatic cancer for patients with hereditary pancreatitis bearing the PRSS1 and the serine protease inhibitor Kazal 1 (SPINK1) variants was estimated to be 40% until the age of 70 years^17^. Selenium exhibits potent anticarcinogenic properties, and its decreased SELENBP1 expression has often been associated with several cancer types. CNDP2 acts as a tumor suppressor, the upregulation of which leads to an activation of the p38 and JNK/MAPK pathways (thereby leading to cell apoptosis), while its downregulated expression results in an activated ERK/MAPK pathway that can promote cell proliferation^18^.

The enriched pathways of the WM7 module significant to POG (Fig. 3B) included the following: (i) the response to chemical stress and the symbiotic process as biological processes (GO); (ii) the carbon metabolism, the tight junction, and the apoptotic pathways as KEGG pathways; and (iii) the disease, the immune system, and the cellular response to stress pathways as Reactome pathways (Fig. S2). The eigen-protein Tu translation elongation factor (EF-Tu) participates in almost all of the mitochondria-mediated protein translation. The upregulation of EF-Tu has been reported in various cancer types, including pancreatic cancers^19^. Hub proteins included the eukaryotic translation elongation factor 2 (EEF2), the HSP90AA1 (Hsp90α), the HSP90AB1 (HSP90β), the 40S ribosomal protein S20 (RPS20), and the 60S acidic ribosomal protein P2 (RPLP2). EEF2 is an essential factor for protein synthesis that has been found overexpressed in numerous types of cancers and that plays an oncogenic role in tumor progression^20^. Both Hsp90α and HSP90β are molecular chaperones that support the folding of newly synthesized proteins and maintain protein stability under various types of cellular stress. An upregulated HSP90α expression has been reported in various types of tumors, including pancreatic cancer^21^. Other key proteins include phosphoglycerate kinase 1 (PGK1), lamin-B1 (LMNB1)), the mitochondrial Lon protease homolog (LONP1), collagen alpha-1(I) chain (COL1A1), and the DNA-dependent protein kinase catalytic subunit (PRKDC). The glycolytic enzyme PGK1 is involved in the HIF1α transcription factor network, in which PGK1 together with pyruvate kinase M2 (PKM2) controls the ATP production during aerobic glycolysis in cancer cells (also known as the “Warburg effect”). The PGK1 overexpression is predictive of poor survival in breast, head and neck, cervical, liver, and pancreatic cancers (*p* < 0.001)^22^. The mitochondrial ATP-dependent protease LONP1 mediates the selective degradation of misfolded, unassembled, or oxidatively damaged polypeptides, as well as certain short-lived regulatory proteins of the mitochondrial matrix. LONP1 reduces mitochondrial stress to promote cell survival, proliferation, and metastasis in cancer^23^. The fibroblast-deriving COL1A1 is involved in extracellular matrix (ECM) remodeling, tumor cell adhesion, and cell migration^24^. The increased expression of COL1A1 can promote cancer progression and metastasis and has been associated with poor prognosis in numerous cancer types^25^. COL1A1 is one of the downstream targets of the glioma-associated oncogenes (GLI1 and GLI2), is involved in collagen deposition, and is associated with PDAC aggression^24^. Finally, PRKDC plays a major role in nonhomologous end-joining DNA repair, which is an important factor for tumor progression and metastasis; in fact, PRKDC is considered an emerging therapeutic target in cancer^26^.

The enriched pathways of the WM11 (green) module significant to the POG (Fig. 3C) included the following: (i) the establishment of localization in cell, transport, and secretion as a biological process (GO); (ii) the carbon metabolism, the biosynthesis of amino acids, and the protein processing in the endoplasmic reticulum as KEGG pathways; and (iii) the disease, the metabolism of proteins, the neutrophil degranulation, the innate immune system, the eukaryotic translation elongation, and the cellular responses to stress as Reactome pathways (Fig. S2). The eigen-protein tropomyosin-1 (TPM1) is an actin-binding cytoskeletal protein, and its altered expression levels have been closely associated with the rearrangement of microfilament bundles that are responsible for the change of cellular morphology and motility. Hub proteins included the elongation factor 1-alpha 1 (EEF1A1), the 40S ribosomal protein SA (RPSA), the elongation factor 1-gamma (EEF1G), the glyceraldehyde-3-phosphate dehydrogenase (GAPDH), the 40S ribosomal protein S3a (RPS3A), the heat shock cognate 71 kDa protein (HSPA8), the endoplasmic reticulum chaperone BiP (HSPA5; GRP78), endoplasmin (HSP90B1), and PKM2. EEF1A1 belongs to the translation factor-related class translation factor GTPase superfamily, and strongly promotes the heat shock response (HSR), thereby protecting cancer cells from proteotoxic stress (such as oxidative stress and hypoxia). An overexpression of EEF1G has been reported in gastric carcinoma, colon adenocarcinoma, and pancreatic cancer^27^. PKM2 is a key regulator of the Warburg effect in cancer cells. Networks of HSP70 or HSP90 (including HSPA8, HSPA5, and HSP90B1) play important roles in the regulation of energy metabolism as well as in cancer cells’ oncogenesis and malignant progression^28^.

The enriched pathways of the WM16 (red) module (Fig. 3D) that are characteristic of the BOG included a symbiotic process, cellular localization, response to the selenium ion, and transport as biological processes (GO) (Fig. S2). The representative eigen- and/or hub proteins included Y-box-binding protein 1 (YBX1), the heterogeneous nuclear ribonucleoprotein D-like (hnRNPDL), the poly(rC)-binding protein 2 (PCBP2; hnRNPE2), the U5 snRNP-specific 200 kDa protein (U5-200KD), the DEAD-box helicase family member DBX (DDX3X), and the small nuclear ribonucleoprotein E (SNRPE); all of which are RNA-binding proteins (RBPs). YBX1 mediates the pre-mRNA alternative splicing regulation and is involved in translational regulation by modulating the interaction between the mRNA and the eukaryotic initiation factors. Moreover, YBX1 is significantly overexpressed in PDAC and has been correlated with poor prognosis and reduced survival. HNRNPDL belongs to the heterogeneous nuclear ribonucleoproteins (representing a large family of RBPs), and its aberrant expression has been reported in several cancer types^29^. PDAC is most prominently characteristic of desmoplasia, an abundant fibrotic stroma in which type I collagen proteins are the most abundant and the main component of the ECM. PCBP2 binds to the C-rich region in 3′-UTR of the collagen α 1(I) mRNA, thereby stabilizing the mRNA and subsequently increasing the type I collagen expression^30^. It has been reported that the combination of gemcitabine with the silencing of PCBP2 can markedly suppress tumor progression in a desmoplastic PDAC orthotopic mouse model^30^. Both ribonucleoproteins U5-200KD and SNRPE are known to engage in the dynamic network of RNA–RNA interactions in the spliceosome machinery. DDX3X is an ATP-dependent RNA helicase that serves multiple functions of cancer (ranging from tumorigenesis to metastasis)^31^ and is involved in many cancer-related pathways (including those of P53, β-catenin, and KRAS)^32^.

### Multivariate correlation analysis (MVA) of key protein expressions

**Figure 4:**
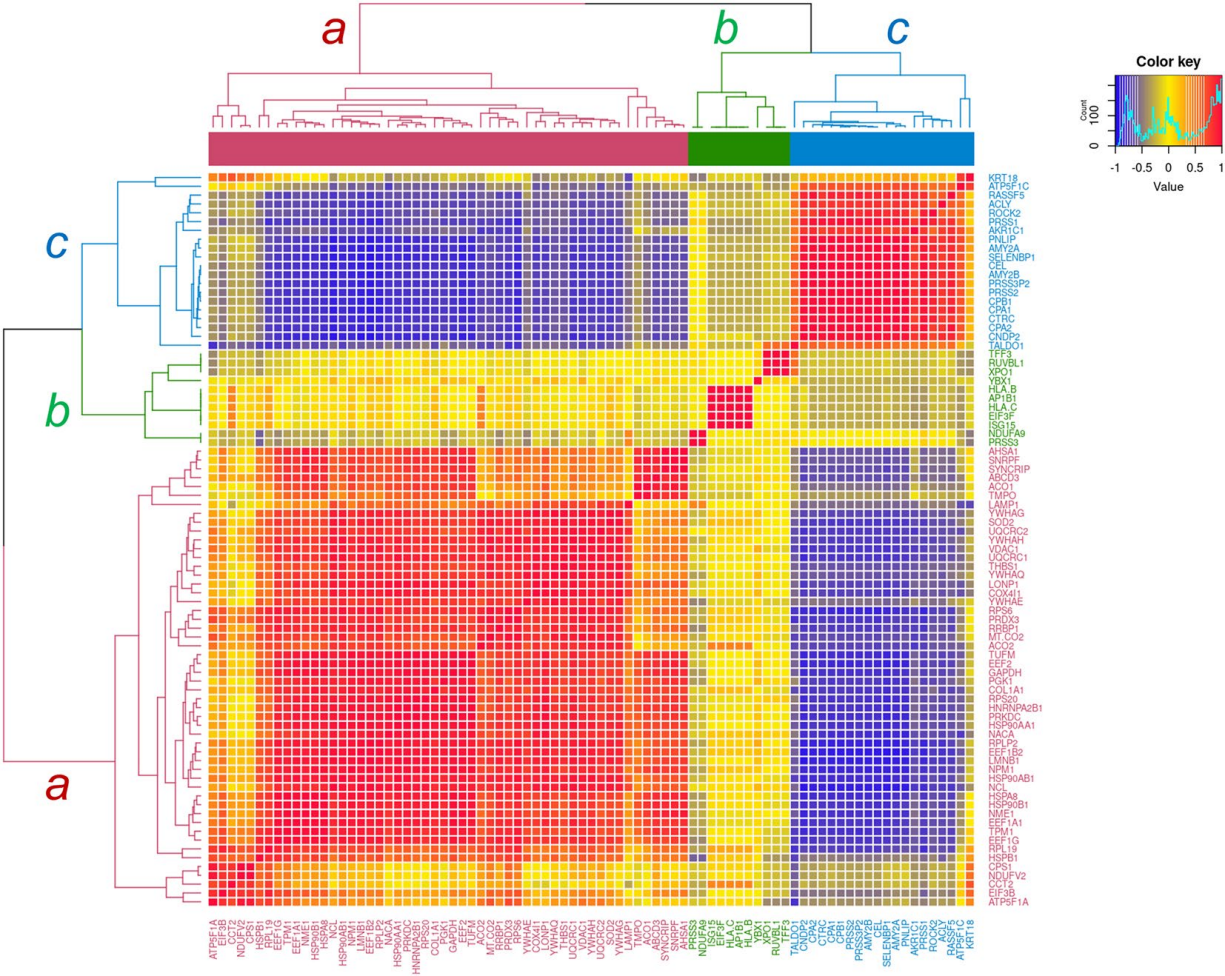
Multivariate correlation analysis (MVA) for the spectral counting-based expression of 87 eigen- and/or hub proteins and other key proteins expressed among all the modules identified for three traits. Clusters are denoted by *a*, *b*, and *c*. Cluster *a* includes the eigen- and hub proteins, and key proteins in the WGCNA module networks correlated to the trait of poor outcome group (POG), Cluster *b* those to the trait of better outcome group (BOG), and Cluster *c* those to the trait of the noncancerous pancreatic duct (NPD).

The representative 87 key proteins expressed in all 18 modules were subjected to an MVA and were clustered into three groups that corresponded successfully to the POG (*a*), the BOG (*b*), and the NPD Group (*c*), respectively (Fig. 4); interestingly, clusters *b* and *c* were found to be close.

### Upstream regulator and causal network analysis by ingenuity pathway analysis (IPA)

The upstream regulator and causal network analysis together with the downstream annotation were performed for the WGCNA modules^33^, where data were analyzed through the use of IPA (QIAGEN Inc., https://www.qiagenbioinformatics.com/products/ingenuitypathway-analysis). Table 2 summarizes the top upstream regulators, master regulators, canonical pathways, and diseases or functions predicted for the four WGCNA modules.

**Table 2:** Representative master regulators predicted to be activated or inhibited (|*z*-value|> 2.0) and upregulated (1.5 < *z*-value < 2.0) are briefly summarized for the four identified WGCNA modules; top annotations of canonical pathways and diseases or functions are also provided.

Module ID (color)	Upstream regulators			Causal networks			Canonical pathways			Diseases or functions		
	Top upstream regulators	z-score	*p* value of overlap	Top master regulators	z-score	Network bias-corrected *p* value	Top 5 annotations	z-score	*p* value	Top annotations	z-score	*p* value
WM5 (greenyellow)	*PTF1A*	1.98	8.67E−08	*NR5A2*	2.24	0.0001	SPINK1 Pancreatic Cancer Pathway	− 2.83	5.01E−24	Synthesis of fatty acid	1.92	0.0096
	*NR5A2*	2.17	4.10E−06	*PTF1A*	2.00	0.0002	Retinol Biosynthesis		0.0002	Fatty acid metabolism	2.16	0.0223
	*GLI1*	− 2.00	0.0163	*CBLC*	− 2.12	0.0024	Triacylglycerol Degradation		0.0003	Morbidity or mortality	− 1.92	0.0240
				*NDRG1*	− 2.84	0.0036	Pulmonary Healing Signaling Pathway		0.0006			
				*NDRG1*	− 2.31	0.0057	SPINK1 General Cancer Pathway		0.0017			
				*plasminogen activator*	2.18	0.0066						
				*SERPINB2*	− 2.18	0.0070						
				*CDC25A*	− 2.12	0.0228						
				*CHEK1*	2.11	0.0272						
				*ALDH3A1*	− 2.07	0.0293						
				*Glycoprotein 1B*	2.07	0.0333						
				*GP9*	2.07	0.0334						
				*MMP8*	2.00	0.0377						
				*GLI1*	− 2.00	0.0395						
				*F3–F7*	2.00	0.0422						
				*RAP2B*	2.36	0.0444						
WM7 (black)	*MYC*	3.31	1.94E−17	*LARP1*	− 2.45	0.0001	Integrin Signaling	1.89	1.91E−06	Cell movement	3.42	7.04E−07
	*Insulin*	2.63	6.28E−13	*CLPP*	− 2.45	0.0001	Paxillin Signaling	2.24	1.20E−05	Migration of cells	3.33	4.38E−06
	*CD3*	2.71	3.15E−09	*MYC*	3.13	0.0001	RHOA Signaling	2.24	2.34E−05	Cell viability	3.10	7.45E−06
	*YAP1*	2.24	3.40E−09	*BRD4*	3.27	0.0001	Actin Cytoskeleton Signaling	2.24	5.62E−05	Invasion of cells	2.96	2.71E−07
	*CLPP*	− 2.45	3.35E−08	*CTDSP1*	− 3.27	0.0001	Epithelial Adherens Junction Signaling	2.24	7.08E−05	Synthesis of protein	2.96	3.65E−08
	*LARP1*	− 2.45	1.58E−07	*CUL4B*	− 2.86	0.0001	Signaling by Rho Family GTPases	2.45	9.33E−05	Organismal death	− 4.38	3.91E−06
	*NFE2L2*	3.16	9.33E−07	*HMGCR*	3.92	0.0001	Fcγ Receptor-mediated Phagocytosis in Macrophages and Monocytes	2.00	0.0001			
	*FGF2*	2.08	1.40E−05	*RND3*	− 3.27	0.0001	Death Receptor Signaling	2.00	0.0001			
	*VEGFA*	2.65	2.81E−05	*RPS14*	− 3.27	0.0001	Regulation of Actin-based Motility by Rho	2.00	0.0003			
	*INSR*	2.62	0.00020	*PFDN5*	− 2.75	0.0001	RHOGDI Signaling	− 2.24	0.0003			
	*STK11*	2.45	0.00023	*FBXL14*	− 3.40	0.0001	MSP-RON Signaling In Cancer Cells Pathway	2.00	0.0006			
	*ANGPT2*	2.17	0.00041	*AMBRA1*	− 2.12	0.0001						
	*IL4*	2.05	0.00053	*FBXO32*	− 3.14	0.0001						
	*MLXIPL*	2.00	0.00090	*CXCL14*	4.00	0.0002						
	*SP1*	1.99	0.00143	*PTPN2*	− 3.18	0.0002						
	*IL5*	2.24	0.00152	*PCGEM1*	2.86	0.0002						
	*CEBPB*	2.41	0.00191	*Mir200*	− 2.54	0.0002						
	*HMGA1*	2.00	0.00204	*VWF*	3.40	0.0003						
	*TGFB1*	2.01	0.00300	*ZEB*	2.20	0.0003						
	*miR-124-3p (and other miRNAs w/seed AAGGCAC)*	− 2.00	0.00465	*Insulin*	2.32	0.0004						
	*PRL*	2.00	0.00925	*USP8*	2.67	0.0007						
	*RICTOR*	− 2.00	0.00970	*LCK/Fyn*	2.31	0.0019						
	*HIF1A*	1.98	0.01660	*SH2D2A*	2.69	0.0019						
	*GLI1*	1.98	0.01950	*CD3*	2.71	0.0030						
	*KLF3*	− 2.00	0.02040	*PIK3C3*	− 2.48	0.0043						
	*STAT3*	2.17	0.04410	*growth factor receptor*	2.99	0.0045						
WM11 (green)	*MYC*	3.11	5.56E−16	*LARP1*	− 2.65	0.0001	Remodeling of Epithelial Adherens Junctions	1.00	1.58E−13	Cell proliferation of tumor cell lines	3.32	2.63E−11
	*CLPP*	− 3.00	9.34E−12	*KDM8*	2.65	0.0001	BAG2 Signaling Pathway	1.00	1.17E−06	Necrosis	− 2.93	1.35E−13
	*PCGEM1*	2.80	9.41E−12	*UBA1*	− 2.45	0.0001	Integrin Signaling	1.89	2.51E−06	Cell death of tumor cell lines	− 2.93	6.75E−10
	*MYCN*	2.21	1.88E−10	*MYCN*	2.14	0.0001	Regulation of Actin-based Motility by Rho	2.24	7.76E−06	Cell survival	2.78	5.77E−09
	*UBA1*	− 2.41	7.00E−10	*CLPP*	− 3.00	0.0001	Actin Cytoskeleton Signaling	1.89	6.31E−05	Cell viability	2.85	2.91E−08
	*KDM8*	2.62	2.90E−08	*PCGEM1*	2.83	0.0001	Fcγ Receptor-mediated Phagocytosis in Macrophages and Monocytes	2.00	0.0006	(Cellular Response to Therapeutics) Sensitivity of carcinoma cell lines	− 2.41	2.44E−06
	*LARP1*	− 2.65	7.75E−08	*MYC*	2.89	0.0001	Leukocyte Extravasation Signaling	2.00	0.0011	Cell viability of tumor cell lines	2.67	5.90E−06
	*XBP1*	2.95	9.67E−08	*CREBZF*	− 3.00	0.0001	RHOA Signaling	2.00	0.0016	Cell death of osteosarcoma cells	− 2.45	1.93E−05
	*IL15*	2.22	1.04E−07	*ZMIZ1*	3.66	0.0001	Synaptogenesis Signaling Pathway	2.24	0.0018	Cell–cell contact	2.93	1.60E−04
	*TGFB1*	2.66	1.47E−06	*Max-Myc*	2.65	0.0001	RHOGDI Signaling	− 1.34	0.0018	Organismal death	− 4.65	2.58E−04
	*IL5*	3.00	4.65E−06	*CUL4B*	− 3.02	0.0001	NAD Signaling Pathway	2.00	0.0033	Apoptosis of colorectal cancer cell lines	− 2.51	0.0008
	*EGFR*	2.00	1.09E−05	*RAD21*	3.02	0.0001						
	*CD3*	2.19	1.99E−05	*SP1-c-Myc*	3.29	0.0001						
	*PRL*	2.75	3.23E−05	*USP10*	− 2.16	0.0001						
	*RICTOR*	− 2.83	3.56E−05	*PCGEM1*	3.29	0.0001						
	*MLXIPL*	2.24	4.45E−05	*H2AX*	3.10	0.0001						
	*SLC13A1*	− 2.24	4.94E−05	*Importin alpha/beta*	− 2.61	0.0001						
	*TSC2*	− 2.22	7.36E−05	*MAP3K12*	3.31	0.0002						
	*HIF1A*	2.22	7.75E−05	*Ep300/Pcaf*	2.29	0.0002						
	*CTNNB1*	− 2.16	9.66E−05	*RASAL1*	− 2.95	0.0002						
	*miR-1-3p (and other miRNAs w/seed GGAAUGU)*	− 2.40	0.0002	*XBP1*	3.00	0.0003						
	*NFE2L2*	2.92	0.0002	*p70 S6k*	2.20	0.0003						
	*ERN1*	2.15	0.0004	*HDAC10*	3.16	0.0004						
	*EGF*	2.18	0.0005	*TRIM28*	− 3.36	0.0006						
	*CD38*	2.22	0.0007	*SIRT7*	− 3.48	0.0008						
	*CSF1*	2.43	0.0010	*TCR*	2.10	0.0008						
	*ESRRA*	2.20	0.0012	*Jmy-p300*	2.45	0.0008						
	*SMAD3*	2.20	0.0015	*miR-483-3p (miRNAs w/seed CACUCCU)*	− 3.61	0.0013						
	*AKT1*	2.24	0.0019	*RNF2*	− 2.29	0.0015						
	(*GLI1*	0.33	0.0005)									
WM16 (red)	*MYC*	1.89	1.86E−05	*PALB2*	− 2.04	0.0010	fMLP Signaling in Neutrophils	2.00	0.0005	Apoptosis of carcinoma cell lines	− 1.89	0.0001
	*CST5*	− 2.65	2.05E−05	*NABP1*	2.20	0.0012	RAC Signaling	2.00	0.0007	Apoptosis of tumor cell lines	− 3.00	0.0003
	*SYVN1*	2.24	7.35E−05	*ZEB1*	2.45	0.0012	RHOGDI Signaling	− 2.00	0.0034	Migration of tumor cell lines	2.63	0.0005
	*IL4*	2.59	0.0002	*ARG2*	− 2.29	0.0027	Senescence Pathway	2.00	0.0105	Cell death of tumor cell lines	− 2.62	0.0005
	*HRAS*	2.19	0.0002	*CD47*	2.12	0.0050				Cell movement of tumor cell lines	2.60	0.0006
	*ATG7*	1.99	0.0002	*TPOR dimer*	2.86	0.0066				Growth of tumor	2.44	0.0007
	*HGF*	2.16	0.0008	*MAP3K12*	2.50	0.0088				Phagocytosis	2.53	0.0013
	*Vegf*	2.45	0.0010	*JINK1/2*	2.04	0.0099				Cell viability	2.31	0.0044
	*JUN*	2.00	0.0041	*CST5*	− 2.65	0.0003				Insulin sensitivity	− 1.98	0.0050
	*SOX2*	2.39	0.0042	*SYVN1*	2.24	0.0013						
	*TP63*	2.17	0.0159									
	*ERBB2*	1.95	0.0239	(*GLI1*	1.18	0.0124)						
	*IGF1*	2.16	0.0352									

#### Upstream and master regulators predicted for the WM5 module

Highly activated upstream and/or master regulators predicted for the WM5 module included *NR5A2*, and *PTF1A*, while *GLI1* was highly inhibited (Table 2). NR5A2 is a nuclear receptor that participates in diverse processes, including bile acid synthesis, the resolution of endoplasmic reticulum stress (ERS), pancreatic development, and acinar differentiation. The pancreas transcription factor 1 alpha (PTF1A) is required for the formation of pancreatic acinar and ductal cells. Gli1 is an oncogenic transcription factor and a critical effector in the Hedgehog pathway and plays a significant role in PDAC progression. The highly inhibited SPINK1 pancreatic cancer pathway (*z* =  − 2.813) was most significantly annotated in the canonical pathway.

#### Upstream and master regulators predicted for the WM7 and WM11 modules

Highly activated regulators for the WM7 protein networks included *MYC*, *BRD4*, *YAP1*, *NFE2L2*, *VEGFA*, *STK11*, *HIF1A*, *SP1*, *STAT3*, and *GLI1*, while *LARP1*, *CLPP*, and *RICTOR* were found to be inhibited (Table 2). The MYC proto-oncogene protein (MYC) binds to the *VEGFA* promoter that promotes the VEGFA production, subsequently leading to angiogenesis. The bromodomain-containing protein 4 (BRD4) binds acetylated histones and plays an important role in epigenetic regulation^34^. YAP1 is the critical transcriptional regulator downstream of the Hippo signaling pathway, while the nuclear factor erythroid 2-related factor 2 (Nrf2 or NFE2L2) is the redox master regulator. The serine/threonine-protein kinase STK11 has been recently suggested to confer protection to cancer cells against metabolic stress and to promote cancer cell survival and invasion, whereas STK11 was previously considered as a tumor suppressor^35^. The hypoxia-inducible factor 1 subunit alpha (HIF1A) is a master transcriptional regulator of the adaptive response to hypoxia. The specificity protein 1 (SP1) is a zinc-finger transcription factor, the overexpression of which has been correlated with poor clinical outcomes in various cancer types, including PDAC. SP1 promotes invasiveness and epithelial-mesenchymal transition (EMT) by cross-talking with STAT3, which in turn regulates pathways of tumorigenesis (including those of tumor cell-cycle progression, apoptosis, angiogenesis, metastasis, and immune system evasion)^36^. Finally, the highly upregulated GLI1 (*z* = 1.98) was indicative of its master regulatory role as a hallmark of PDAC^37^.

By contrast, the La-related protein 1 (LARP1) was found to be highly inhibited. LARP1 is the master regulator of the cap-dependent Top mRNA translation; thereby, our findings strongly suggest the inhibition of protein synthesis via cap-dependent mRNA translation or the activation of the cap-independent, IRES-mediated translation of mRNA subsets that encode oncogenic proteins (including HIF1α, MYC, and VEGFA)^38^. Caseinolytic protease P (CLPP) plays a key role in the mitochondrial unfolded protein response and is linked to the regulation of cellular bioenergetics^39^.

Highly activated regulators predicted for the WM11 protein networks included *MYC*, *PCGEM1*, *MYCN*, *KDM8*, *XBP1*, *TGFB1*, *HIF1A*, *NFE2L2*, *ERN1*, *CD38*, *CSF1*, and *SMAD3*, and highly inhibited regulators included *LARP1*, *CLPP*, and *RICTOR*. Thus, both WM7 and WM11 modules share several key regulators (Table 2). The prostate cancer gene expression marker 1 (*PCGEM1*) does not code for a protein, but it is a long noncoding RNA PCGEM1 (lncRNA PCGEM1). Hypoxic cancer cells contain large amounts of exosomal lncRNA PCGEM1, which plays a crucial role in proliferation, migration, invasion, drug resistance, and angiogenesis in cancer^40^. The deregulation of MYCN (N-MYC), as well as of other MYC family oncogenes, is frequently associated with a poor prognosis in many types of cancer. KDM8 is a histone lysine demethylase/dioxygenase that demethylates H3K36me2 by inducing an epigenetic dysregulation that is implicated in carcinogenesis. The oncogenic histone demethylase KDM8 has also been reported to form a partnership with PKM2, thereby promoting PKM2 nuclear translocation^41^. The X-box binding protein 1 (XBP1) plays a key role in the unfolded protein response (UPR) under ERS^42^. The ER stress response element is present in the promoter region of HSPA8 (Grp78 or BiP; the master regulator of ER stress) and has been captured by the data-driven WM11 protein networks (Fig. 3C). *ERN1* encodes the endoplasmic reticulum-to-nucleus signaling 1, known as inositol requiring enzyme 1 (IRE1α), and the IRE1α/XBP1 pathway is one of the major UPR pathways and the most highly conserved ERS pathway^43^. The expression of CD38 is involved in tumor cell escape from the PD-1/PD-L1 blockade^44^ and can be upregulated in response to a PD-L1 antibody therapy^45^.

PDAC is characterized by dense desmoplasia in which the fibrotic stroma contains a high number of activated pancreatic stellate cells (PSCs). The aggressive nature of PDAC is now attributed to cells capable of interplaying the surrounding ECM of the tumor microenvironment to promote disease progression and resistance to therapy. Recently, Steins et al. have shown that PDAC cells induce the secretion of colony-stimulating factor 1 (CSF1), which deactivates stromal PSCs, thereby promoting the development of aggressive subtypes of pancreatic tumors^46^. The EMT-like features are often characteristic of high-grade PDACs. PDAC patients usually harbor SMAD4 mutations and deletions; in such cases, SMAD3 is found to be accumulated in the nucleus, and its upregulation has been correlated with the EMT-like features seen in PDAC, regardless of the SMAD4 status^47^.

#### Upstream and master regulators predicted for the WM16 module

Highly activated regulators for the WM16 protein networks included *MYC*, *SYVN1*, *HRAS*, *hepatocyte growth factor (HGF)*, and *SOX2*, while highly suppressed regulators included *CST5* and *PALB2*. Interestingly, GLI1 was greatly restricted (*z* = 1.18) (Table 2). Synoviolin 1 (SYVN1) is a transmembrane E3 ubiquitin-protein ligase that accepts ubiquitin specifically from the ER-associated ligase and transfers it to substrates, thereby promoting their degradation. Cancer cells harboring a p53 mutation (~ 70% of PDAC cases) are resistant to anticancer chemotherapy and are characterized by aggressive phenotypes. p53 regulates the ER function in response to stress. Namba et al. have demonstrated that the p53 function loss upregulates IRE1α that subsequently targets XBP1^48^. The latter enhances the activation of the IRE1α-XBP1 pathway, thereby providing a response to ERS and unfolding protein stress^48^. The ER membrane protein homeostasis is maintained by ER-associated degradation^49^, while SYVN1 promotes the ubiquitination and degradation of IRE1α. Moreover, SYVN1 is a favorable prognostic marker in head and neck cancer (*p* < 0.001) (https://www.proteinatlas.org). Inhibiting the activation of the IRE1α/XBP1 pathway to maintain the ER function through an SYVN1-dependent proteasomal degradation of IRE1α could be a promising modality for treating cancer cases lacking the p53 function^48^.

Oncogenic KRAS mutations are predominant in PDAC (Fig. S3)^50^ and are thought to be the driver mutations in PDAC*.* However, two other RAS family members, NRAS and HRAS, can be activated in PDAC by the oncogenic KRAS. Weyandt et al. have shown that the loss of wild-type HRAS increases tumor load and reduces the survival in an oncogenic KRAS-driven PDAC mouse model^51^. They have also examined those results by tracing the earliest stages of pancreatic cancer and have suggested that the wild-type HRAS is tumor suppressive during these early stages^51^. The paracrine HGF is strongly secreted from PSCs. Yan et al. have shown (by using the pancreatic cancer cell-line Panic-1) that the paracrine HGF (via its receptor c-MET activation) can induce a YAP nuclear translocation and an HIF1α stabilization, thereby promoting the expression of cancer stem cell pluripotency markers (including the sex-determining region Y (SRY)-Box2; SOX2) and tumorsphere formation^52^. Xu et al. have reported that the paracrine HGF can also activate the c-MET/PI3K/AKT pathway to induce the EMT and inhibit the apoptosis in pancreatic cancer cells, thereby enhancing gemcitabine chemoresistance^53^.

SOX2 is a key regulator of cancer stemness in PDAC^54^. Interestingly, Wuebben et al. have demonstrated that inducible overexpression of SOX2 in engineered PDAC cell lines inhibits growth in vitro and reduces tumorigenicity, while an inducible knockdown of SOX2 has been shown to reduce the PDAC growth both in vitro and in vivo^55^. SOX2 functions seem to act as a molecular rheostat for the control of the growth, tumorigenicity, and drug response of PDAC cells, while the latter seems to highly depend on the expression of optimal levels of SOX2^55^. Thus, the activation of SOX2 (*z* = 2.39) predicted for the BOG in this study might imply that the overexpression of SOX2 restricts the growth of PDAC cells, while the endogenous intermedium levels of SOX2 lead to maximum tumor growth.

Interestingly, the inhibition of both the CST5 and the PALB2 was predicted for the BOG. p53 directly induces cystatin-D (CST5), which functions (in most cases) as a tumor suppressor by promoting the mesenchymal-epithelial transition^56^. Contrary to expectations, the survival analysis of the CST5 mRNA expression data of pancreatic cancer patients (*n* = 176) from The Cancer Genome Atlas (TCGA) database indicated that the high CST5 expression can be correlated with an unfavorable overall survival rate (*p* < 0.05) (https://www.proteinatlas.org), which might suggest that CST5 acts as a tumor-promoting factor in pancreatic cancer.

The partner and localizer of BRCA2 (PALB2) plays a critical role in homologous recombination repair and is also considered a susceptibility gene for pancreatic cancer^57^. Ge et al. have demonstrated that the PALB2‑knockdown can significantly decrease PDAC cell migration (but not cell proliferation) and that the overall survival is negatively correlated with the PALB2 expression^58^. We performed a web-based survival analysis (KMplot) for the Pan-cancer mRNA RNA-seq data of PDAC (*n* = 177) of the TCGA database and confirmed a significant negative association between the high PALB2 expression and the overall survival (log-rank test *p* = 2.7 × 10^−5^; hazard ratio: 2.38) (Fig. S4)^59^.

A PALB2 mutation is expected to disrupt the BRCA1 and BRCA2 interactions that are critical to DNA double-strand break repair. Global genomic sequencing has identified a biallelic inactivation of PALB2 in a patient who had advanced gemcitabine-resistant pancreatic cancer; the patient later received mitomycin C (a DNA damaging agent) based on a personalized therapy and responded well for 36 + months (when the expected median survival was 3 months)^60^. Poly(ADP-ribose) polymerases (PARPs) are DNA damage sensors and key regulators of single-stranded DNA break repair. Clinical and preclinical studies of talazoparib, a PARP inhibitor (PARPi), delivered to PALB2-deficient solid tumors have suggested that the PARPi can exert a synthetic lethal effect in PALB2-deficient tumors, thereby recommending that the *PALB2* status should be assessed for securing the best clinical outcome for a patient^61^.

## Discussion

The WGCNA analysis following the MS-based proteomic analysis has identified protein co-expression networks that are significantly and characteristically associated with the POG and the BOG. The MVA for key proteins has successfully exhibited three clusters that corresponded to the three traits studied (Fig. 4).

**Figure 5:**
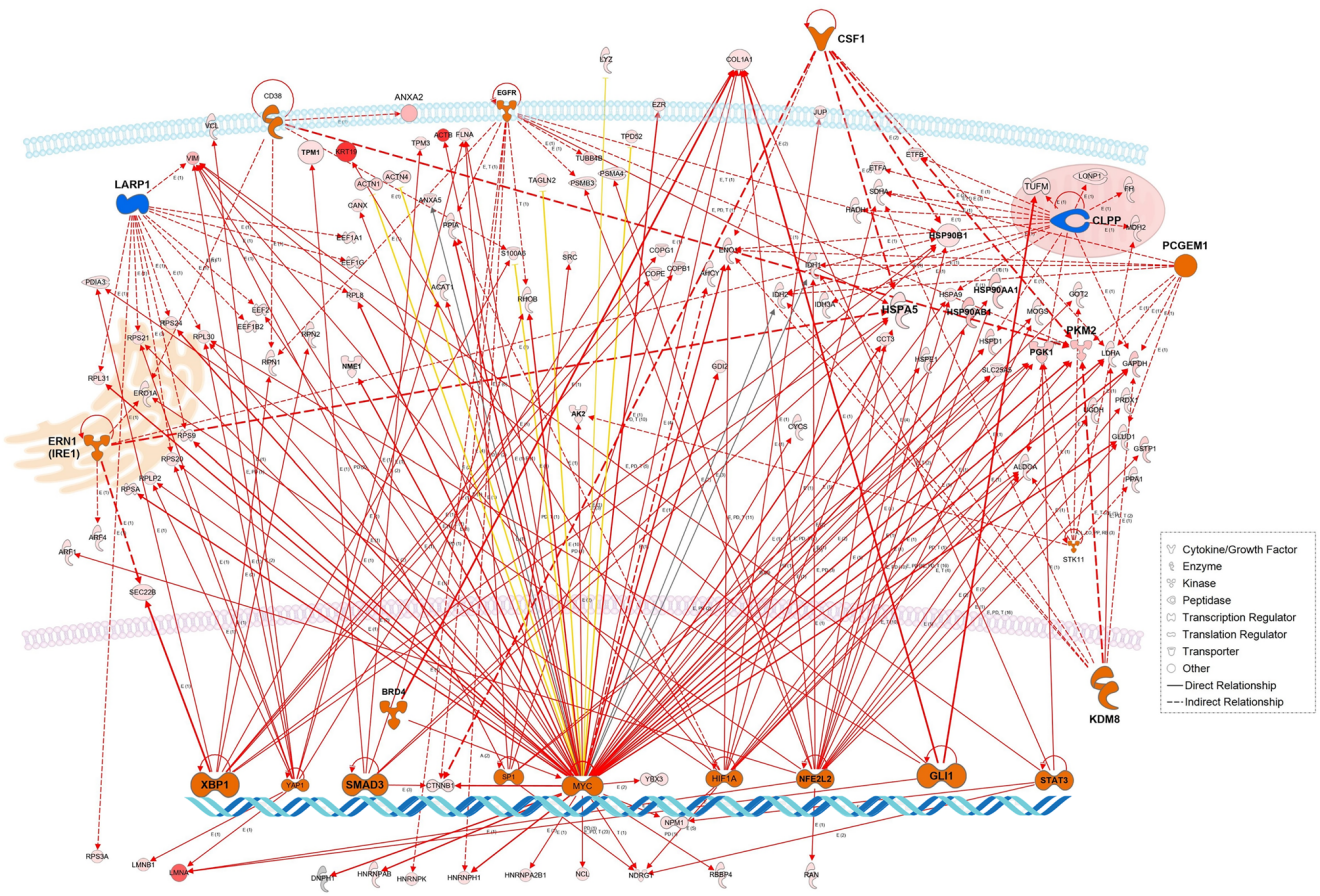
Integrative network of representative upstream and master regulators, along with their target molecules in datasets, as predicted for the WM11 and WM7 modules (significant to the poor outcome group; POG).

Oncogenic KRAS induces the redox master regulator NFE2L2, followed by activation nf HIF1A, MYC, and MYCN. Both the WM7 and WM11 protein networks were mainly associated with activation of the HSR pathways, the witching to an IRES-mediated mRNA translation, the Warburg effect, and the UPR under hypoxia-related stress and ERS. *GLI1*, *NFE2L2*, and *HIF1A* were identified as key regulators of the WM7 module. GLI1 and the highly activated STAT3 and BRD4 imply the involvement of a non-canonical Hedgehog pathway. Those of the WM11 module, besides NFE2L2 and HIF1A, include lncRNA *PCGEM1*, *KDM8*, *XBP1*, *ERN1* (IRE1α), *CD38*, and *CSF1*. The activation of both XBP1 and IRE1α, together with the upregulated Grp78, strongly suggests an upregulated unfolding protein response via the IRE1α/XBP1 pathway. The aggressive natures of pancreatic tumors are associated with the deactivation of stromal PSCs induced by CSF1 as well as with the mesenchymal nature of PDAC cells induced by SMAD3. An integrative network of representative upstream and master regulators, along with their target molecules in datasets, as predicted for both the WM11 and WM7 modules is presented in Fig. 5.

**Figure 6:**
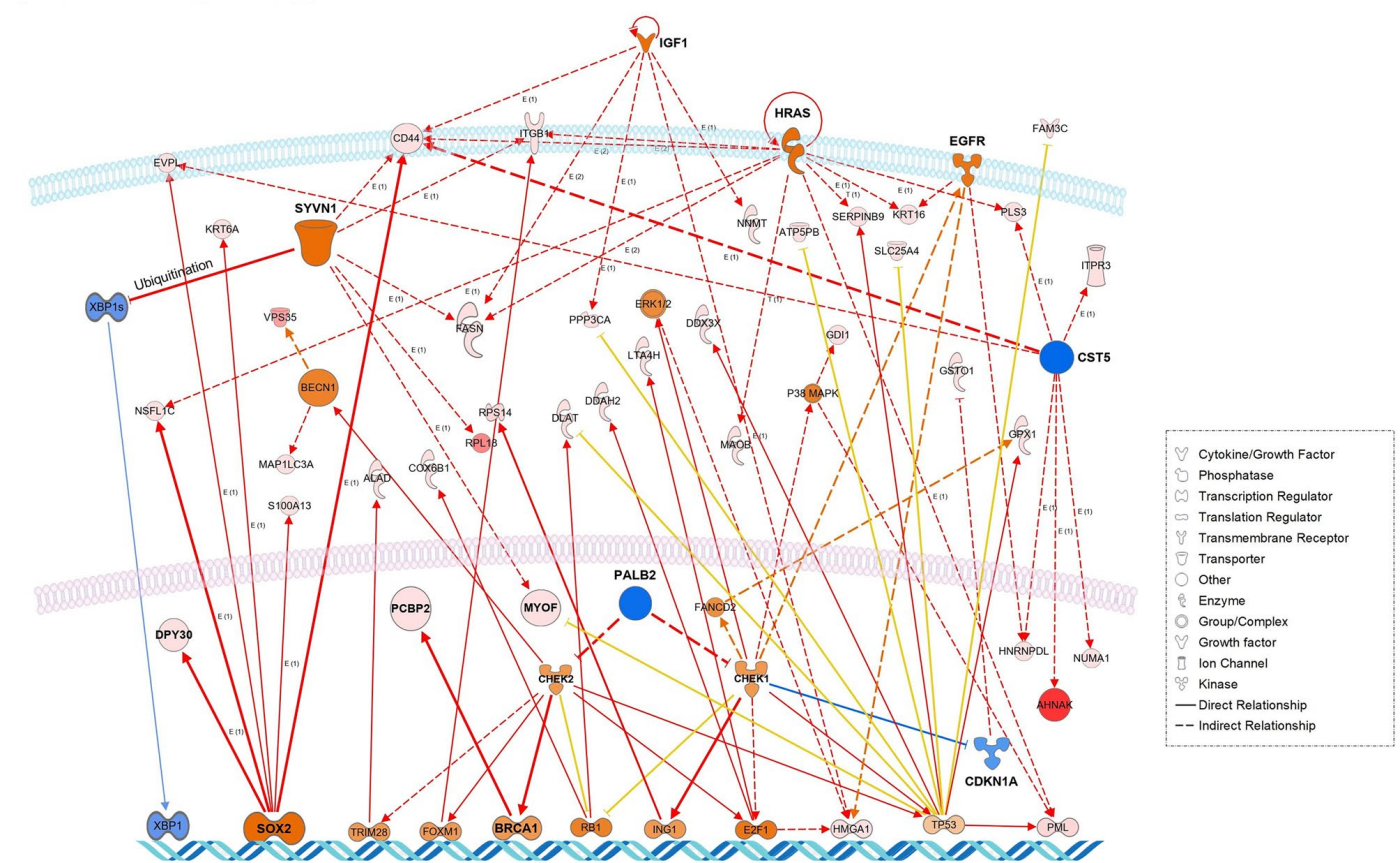
Integrative network of representative upstream and master regulators, along with their target molecules in datasets, as predicted for the WM16 module (characteristic of the better outcome group; BOG).

The WM16 (red) module was representative of the BOG. Lack of p53 function (due to its frequent mutations) induces the expression of IRE1α, and subsequently, the signaling pathway of the IRE1α/XBP1 axis is activated to respond to the ER stress (that contributes to the malignant phenotypes, including those of chemoresistance and metastasis). The association of IRE1α with SYVN1 leads to the degradation of IRE1α, which promotes ER organelle homeostasis. Highly activated levels of SOX2 might also induce the reduction of tumor growth through its molecular rheostat function. Moreover, the highly inactivated PALB predicted for BOG suggests that the loss of PALB function might be also associated with the suppression of malignant tumor progression and/or chemoresistance; a suggestion that remains to be clarified. An integrative network of representative upstream and master regulators, along with their target molecules in datasets, as predicted for the WM16 module is presented in Fig. 6.

The limitation of this study is the number of patients examined due to the only eight cases available in our hospital, which were with the same histological IIB grade, with the same clinical stage, and treated similarly but resulted differently in better or poor outcomes. We plan to verify the results of this study by using a larger sample size of the external cohort being accumulated in the future.

In conclusion, we have successfully applied WGCNA to clinical proteomics datasets. Our results revealed data-driven co-expression networks and their upstream and master regulators associated with the poor and better outcome PDAC groups. Considering that a limitation of this study was the limited number of patients examined, a future larger-sample cohort study that would include a genomic alteration analysis and investigate data-driven proteogenomic networks might provide even more clinically meaningful insight into the proteogenomic landscape of PDAC.

## Materials and methods

### PDAC FFPE tissue specimens and sample preparation

This study was approved by the Tohoku University Ethics Committee (2006-119). The FFPE tissues were obtained from individual patients along with their informed consent, and the study adhered to the Helsinki Declaration. Resected pancreatic tissues were fixed in 4% paraformaldehyde and routinely processed for paraffin sectioning. For tissue microdissection, 10-μm-thick sections from the FFPE tumor blocks were cut and placed on DIRECTOR™ slides (OncoPlex Diagnostics Inc., Rockville, MD, USA). The sections were then deparaffinized and stained with hematoxylin by using standard histological methods before dissection. Microdissection was performed by using a Leica LMD6000 (Leica Microsystems GmbH, Wetzler, Germany). A total area of 8 mm^2^ (with approximately 30,000 tumor cells) was directly transferred from the FFPE sections, via laser dissection, into the cap of a 200-μL low-binding PCR tube. Proteins were extracted and digested with trypsin by using the Liquid Tissue™ MS Protein Prep kits (OncoPlex Diagnostics Inc.) according to the manufacturer’s instructions^62^. Briefly, dried microdissection pellets were suspended in 20 μL of Liquid Tissue buffer and heated at 95 °C for 90 min, and then cooled on ice for 3 min before the addition of 0.1 μg of trypsin to each tube. The tubes were then incubated at 37 °C overnight. Dithiothreitol was added to a final concentration of 10 mM, and the samples were heated for 5 min at 95 °C. The digested samples were dried, then resuspended in 50 μL of a 2% acetonitrile aqueous solution containing 0.1% trifluoroacetic acid and stored at − 20 °C until analysis. The pathological specimens were independently reviewed by two pathologists (S. M. and M. U.).

### Global proteomics by LC–MS/MS

A label-free spectral counting-based quantitative proteomic analysis was conducted through LC–MS/MS^63^. The digested samples (5 μL for a single run) were analyzed in triplicate by LC–MS/MS by using a reverse-phase LC interfaced with an LTQ-Orbitrap hybrid MS (Thermo Fisher Scientific, CA) and a nano-electrospray ionization device (AMR Inc., Tokyo, Japan). The LC system consisted of Paradigm MS4B (Michrom BioResources, CA), a trap cartridge (0.3 mm × 5.0 mm, CERI, Tokyo, Japan), a peptide Cap-Trap cartridge (2.0 × 0.5 mm^2^ inside diameter), and an analytical column (L-column Micro, 150 × 0.2 mm^2^ L-C18, 3 μm, 12 nm; Chemical Evaluation Research Institute, Tokyo, Japan) fitted with an emitter tip (FortisTip, OmniSeparo-TJ, Hyogo, Japan). An aliquot of samples was loaded into the trap and was then washed with solvent A (2% acetonitrile aqueous solution containing 0.1% formic acid) to concentrate the peptides in the trap and desalt them. Subsequently, the trap was connected to the separation column, and the peptides were eluted from the whole column with a 0.1% formic acid aqueous solution and acetonitrile, by a linear 5–40% acetonitrile concentration gradient over 70 min, at a flow rate of 1 μL/min.

### LC–MS/MS analysis

All MS/MS spectral data were searched against the *Homo sapiens* entries in the Swiss-Prot database (Release 57.13, 20,349 entries) by using the Mascot software (version_2.1.1; Matrix Science, London, UK). This search considered tryptic peptide candidates, and the formylation of lysine and the oxidation of methionine were considered variable modifications. The peptide mass tolerance was 20 ppm, the fragment mass tolerance was 0.8 Da, and the trypsin specificity was applied with a maximum of two missed cleavages. A *p* value lower than 0.05 was considered to be statistically significant in the protein identification^64^. The expressions of the identified proteins were assessed by spectral count-based protein quantification. The spectral count is the number of MS/MS spectra assigned to each protein.

### WGCNA, a weighted correlation network analysis

The similarity in protein expression patterns for all protein pairs was calculated according to their pairwise Pearson’s correlation coefficient; i.e., the similarity between proteins i and j was defined as (1 − *r*_i,j_)/2, where *r*_i,j_ is the Pearson’s correlation coefficient of the protein expression patterns between these two proteins. We performed a network topology analysis for various soft-thresholding powers ranging from 1 to 100 to choose an optimal value of balance between independence and mean connectivity. A topological overlap matrix (TOM) that considers topological similarities between a pair of proteins in the network was then generated from the resultant scale-free co-expression network. We generated a tree that clustered proteins in its branches by hierarchical clustering through the use of dissimilarity according to TOM (1 − TOM), and protein modules were determined by using a dynamic tree-cutting to trim the branches^9^.

The modules that were summarized by the first principal component are referred to as eigen proteins in the text, as they express the highest connectivity in the module. Module membership, defined as the correlation between the protein expression profile and the module eigen-protein, was measured with values ranging from 0 to 1, with “0” representing a gene that is not part of the module and “1” representing high connectivity with the module. Subsequently, the module-trait association was determined by using the correlation between the module eigen-protein and the three clinical traits: the POG, the BOG, and the NPD group. A protein module was summarized by the top hub protein (referred to as “eigen-protein”) with the highest connectivity in the module. The WGCNA analysis was performed using the WGCNA R-package^9^ that is implemented in RStudio.

### Protein-PPI network construction

We used the STRING database (version 11.5) to construct a PPI network for a protein module^12^. STRING networks were calculated under the criteria for linkage with experiments, databases, text mining, and co-expression, by using the default settings (medium confidence score: 0.400; network depth: 0 interactions). Functional enrichment results were obtained for canonical pathways, with a *p* < 0.05. Proteins in a protein module were mapped in the PPI network from the STRING database to produce the results of the enrichment analysis regarding the biological process (GO), the KEGG pathways (hsa), and the Reactome pathways (HAS). Protein networks were subsequently exported to Cytoscape (version 3.8.2)^13^ from the STRING database. We then identified the hub proteins in each module according to their intramodular connectivity, and their correlation with module eigen proteins. The proteins inside the co-expression modules exhibit high connectivity, and the proteins within the same module may play similar roles. The top 10 high-degree proteins were identified by using the *cytoHubba* plugin^14^. The top-ranked proteins in each module were considered to be hub proteins; hereby referred to as “highly connected proteins.” Functional enrichment results were obtained for canonical pathways by considering a *p* value of < 0.05 to be statistically significant.

The multivariate correlation analysis (MVA) of semiquantitative key protein expressions was performed by using the JMP software (SAS Institute, Cary, NC, USA), and which result was visualized using Intervene Shiny App (https://intervene.shinyapps.io/intervene/)^65^.

### Upstream regulator and causal network analysis by IPA

Upstream regulators, causal networks, and canonical pathways were predicted by employing the IPA software (http://resources.qiagenbioinformatics.com/getting-started-guides/citing-ingenuity-products.pdf)^33^. Quantile-normalized protein expression data of the selected modules were used as input datasets. Both the upstream regulators and causal networks (*p* < 0.05) predicted from the WGCNA network modules were significantly associated with the three clinical traits (POG, BOG, and NPD group), where the activation and the inhibition of a predicted network were defined by *z*-values that were > 2.0 and <  − 2.0, respectively. The upregulation was defined by *z*-values being > 1.5 and < 2.0, while the downregulation was defined by *z*-values being >  − 2.0 and <  − 1.5.

## Supplementary Information

Supplementary Information.

## Data Availability

The unfiltered MS datasets generated and analyzed in this study have been deposited in the ProteomeXchange (http://proteomecentral.proteomexchange.org) and jPOST (https://repository.jpostdb.org), with the dataset identifiers PXD032681 and JPST001535, respectively.
