# Supplementary material for: Disease-related protein co-expression networks are associated with the prognosis of resectable node-positive pancreatic ductal adenocarcinoma
Source: Sci Rep. 2022 Aug 29;12:14709. doi: 10.1038/s41598-022-19182-9 (PMC9424258; doi:10.1038/s41598-022-19182-9)

Supplementary Information File 1

Disease-related protein co-expression networks are associated with the prognosis of resectable node-positive pancreatic ductal adenocarcinoma

Toshihide Nishimura, Tatsuyuki Takadate, Shimpei Maeda, Takashi Suzuki, Takashi Minowa, Tetsuya Fukuda, Yasuhiko Bando, and Michiaki Unno

Figure S1. The relationship between module eigen-proteins and NPD, POG, and BOG clinical traits.

The first and second columns in the table represent the module identification and color name of the module, respectively. The third column represents the number of proteins in each module. The fourth to sixth columns are color-coded by the correlation coefficient according to the color legend on the right side of the seventh column, in which the intensity and direction of the correlations are indicated (red, positive correlation; blue, negative correlation). The *p*-values of the correlation coefficients and *q*-values by multiple testing correction using the Benjamini–Hochberg (BH) method are presented. The fourteenth to seventeenth columns present the BH-corrected *q*-value by the hypergeometric overlapping test and maximum *q*-value. The eighth to seventeenth columns with significant *p*- and *q*-values (< 0.05) are highlighted in the bright red background.

Module ID	Module color	# proteins	***Correlation* (*r*)**			***p*-value**			**BH-corrected *q*-value [a)]**			**Hypergeometric overlapping BH-corrected *q*-value [a)]**			**Max. *q*-value**
			NPD	***POG***	***BOG***	***NPD***	***POG***	***BOG***	***NPD***	***POG***	***BOG***	***NPD***	***POG***	***BOG***	
WM1	light-green	21	0.44	-0.23	-0.24	0.133	0.460	0.433	0.342	0.637	0.866	4.416E-07	1.0000	1.0000	4.416E-07
WM2	light-cyan	24	0.39	-0.2	-0.21	0.190	0.514	0.492	0.380	0.544	0.805	8.725E-18	1.0000	1.0000	8.725E-18
WM3	salmon	38	0.46	-0.3	-0.19	0.110	0.319	0.537	0.330	0.574	0.744	1.409E-11	1.0000	1.0000	1.409E-11
WM4	grey60	23	0.47	-0.2	-0.26	0.107	0.451	0.383	0.482	0.677	0.985	2.340E-12	1.0000	1.0000	2.340E-12
WM5	green-yellow	42	0.88	-0.43	-0.51	6.070E-05	0.146	0.078	0.001	0.329	1.402	2.477E-12	1.0000	1.0000	2.477E-12
WM6	pink	47	0.47	-0.26	-0.23	0.108	0.395	0.443	0.389	0.646	0.797	6.446E-24	1.0000	1.0000	6.446E-24
WM7	black	66	-0.56	0.76	-0.16	0.046	0.003	0.591	0.275	0.025	0.665	1.0000	1.714E-03	1.0000	1.714E-03
WM8	turquoise	179	-0.35	0.54	-0.17	0.240	0.059	0.588	0.432	0.212	0.706	1.0000	1.426E-04	1.0000	1.426E-04
WM9	brown	130	-0.28	0.41	-0.12	0.359	0.163	0.700	0.462	0.326	0.700	1.0000	0.0030	1.0000	0.0030
WM10	cyan	38	-0.42	0.6	-0.15	0.151	0.031	0.616	0.340	0.183	0.652	1.0000	0.2571	1.0000	0.2571
WM11	green	98	-0.62	0.94	-0.29	0.024	1.420E-06	0.336	0.217	2.56E-05	1.008	1.0000	1.621E-06	1.0000	1.621E-06
WM12	blue	163	-0.27	0.46	-0.17	0.363	0.111	0.570	0.436	0.285	0.733	1.0000	5.625E-17	1.0000	5.625E-17
WM13	tan	40	-0.31	0.58	-0.25	0.304	0.038	0.403	0.497	0.171	0.907	1.0000	1.0000	1.0000	1.0000
WM14	yellow	114	-0.28	0.49	-0.19	0.352	0.089	0.525	0.487	0.266	0.788	1.0000	2.834E-04	1.0000	2.834E-04
WM15	purple	46	-0.24	-0.22	0.48	0.421	0.471	0.099	0.446	0.565	0.446	1.0000	1.0000	1.0512E-20	1.051E-20
WM16	red	68	-0.25	-0.22	0.49	0.404	0.467	0.091	0.455	0.600	0.543	1.0000	1.0000	3.485E-39	3.485E-39
WM17	magenta	47	-0.3	-0.18	0.50	0.316	0.555	0.083	0.474	0.555	0.743	1.0000	1.0000	1.961E-08	1.961E-08
WM18	Midnight-blue	34	-0.2	-0.21	0.42	0.518	0.485	0.152	0.518	0.546	0.547	1.0000	1.0000	9.636E-17	9.636E-17

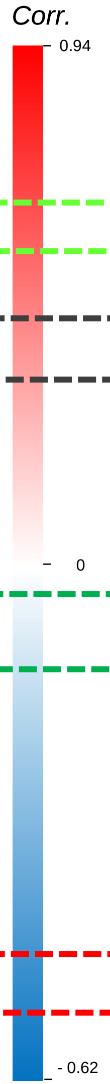

Note [a)] multiple testing correction using the Benjamini–Hochberg method.

Figure S2. Pathway enrichment results obtained for the WGCNA modules. Among pathways enriched for the protein core networks obtained for biological process (GO), KEGG pathways, and Reactome pathways, top 10 pathways are presented in the order of significance by *q*-value.

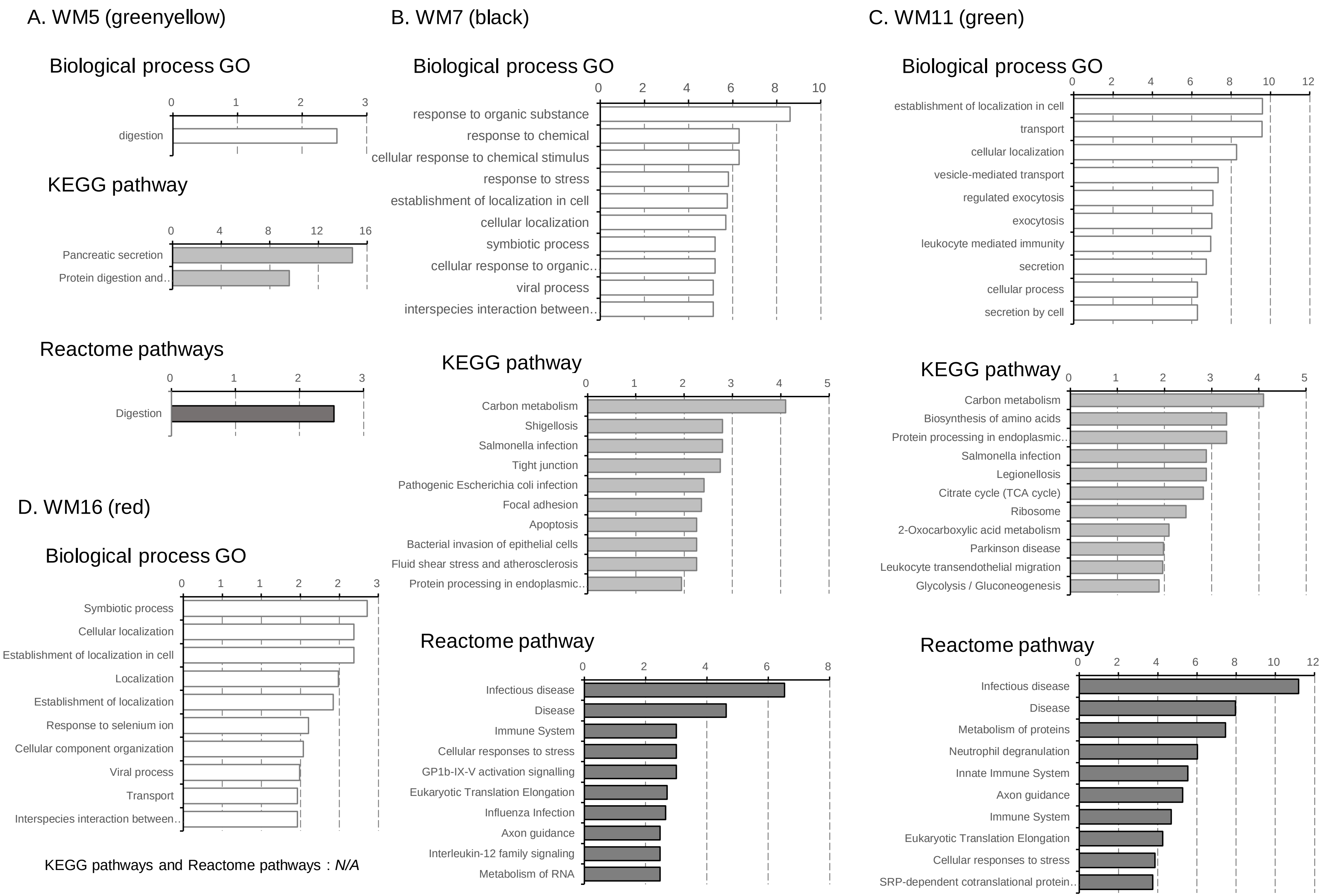

Figure S3. Genomic alteration landscape of pancreatic ductal adenocarcinoma (CPTAC, Cell 2021) (140 patients/samples) [49].

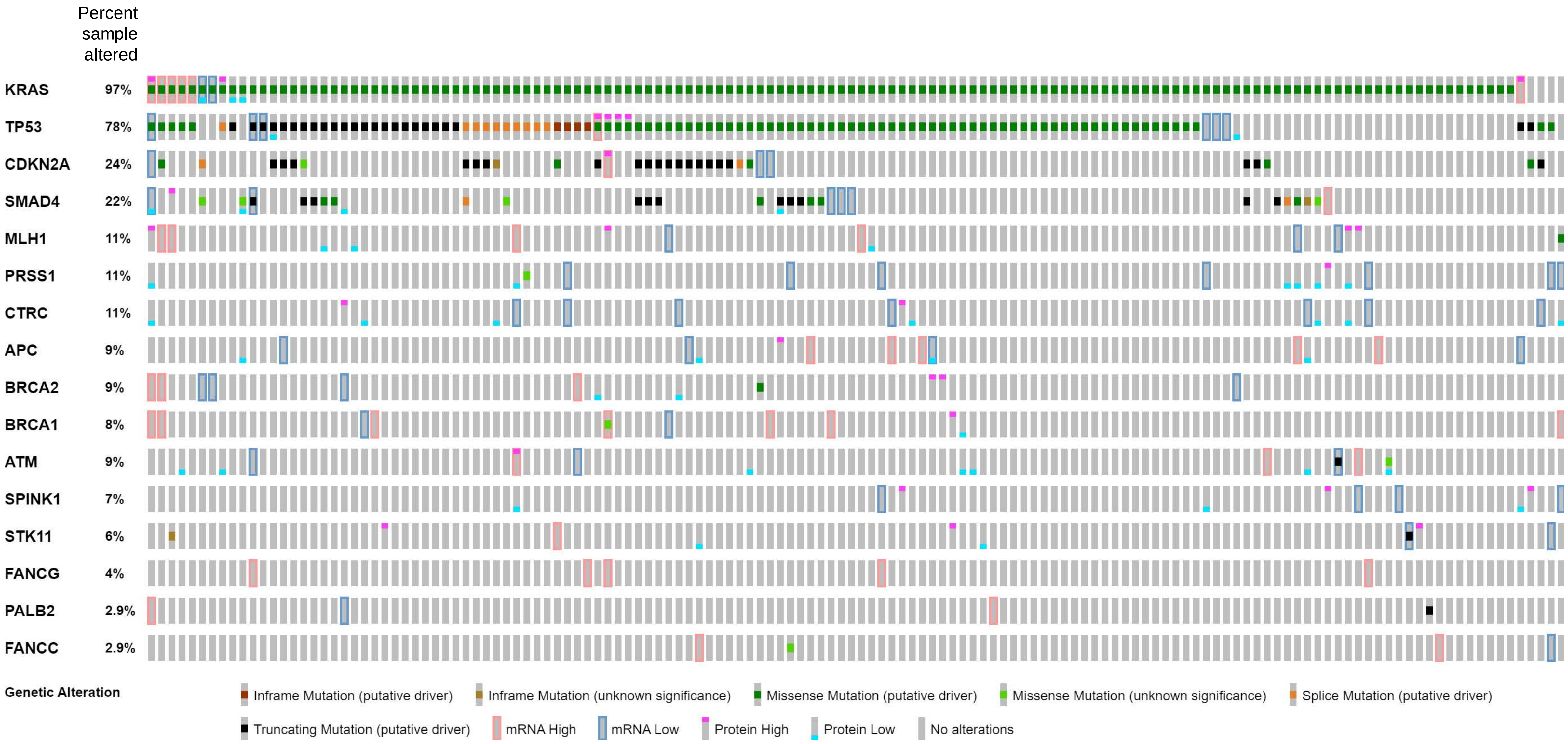

Figure S4. the web-based survival analysis (KMplot) of PALB2 for the Pan-cancer mRNA RNA-seq data of PDAC (n = 177) from the TCGA database [58].

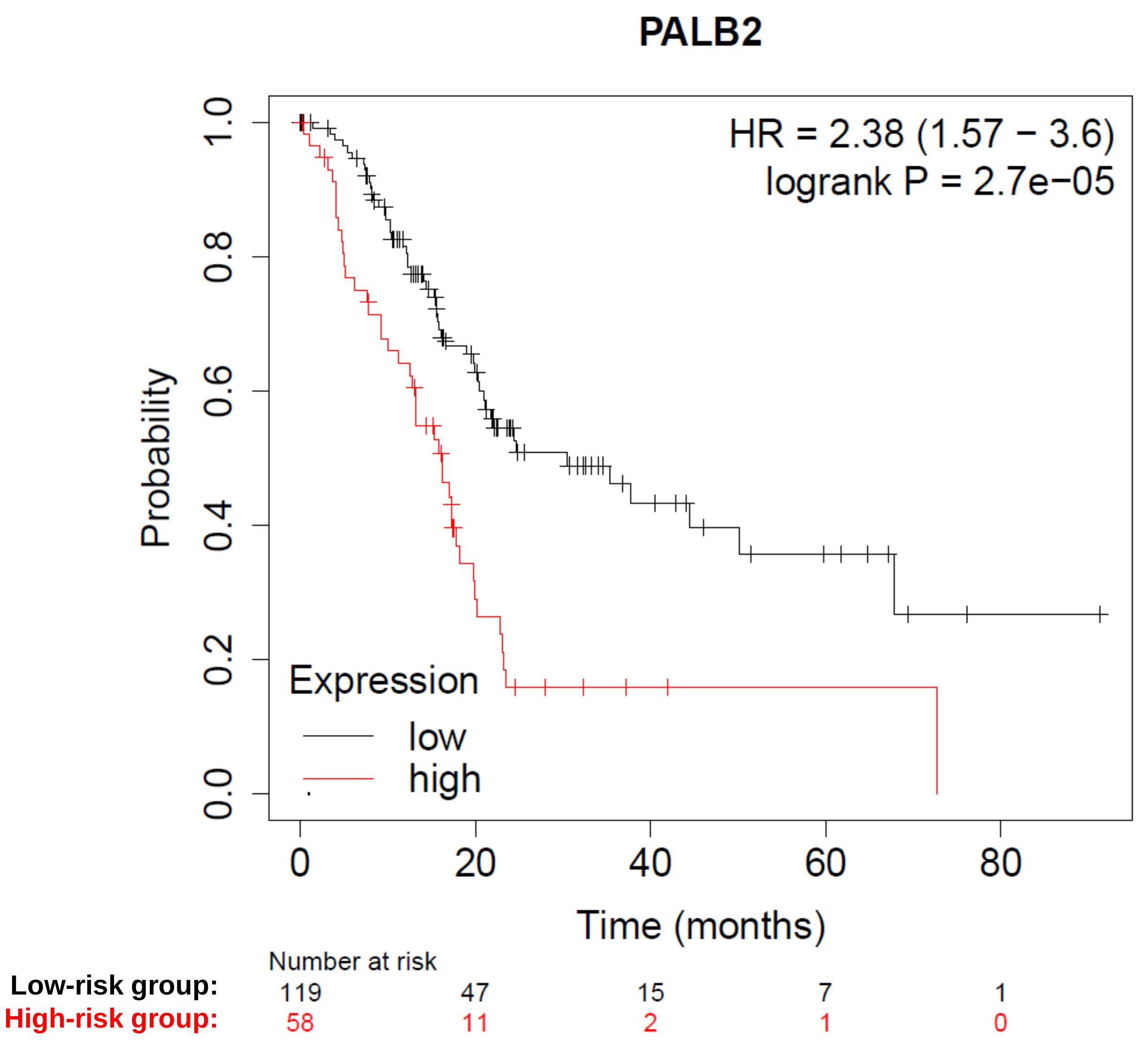

Supplement: Supplementary file 1 — Supplementary Information. [file 41598_2022_19182_MOESM1_ESM.pdf]
